# Prevalence and Characterization of Shiga Toxin Producing *Escherichia coli* Isolated from Animal Feed in Croatia

**DOI:** 10.3390/microorganisms10091839

**Published:** 2022-09-14

**Authors:** Marijana Sokolovic, Borka Šimpraga, Tajana Amšel-Zelenika, Marija Berendika, Fani Krstulović

**Affiliations:** Croatian Veterinary Institute, Poultry Centre, Heinzelova 55, 10000 Zagreb, Croatia

**Keywords:** *Escherichia coli*, STEC, feed, poultry, *stx*, *eae*

## Abstract

A survey on prevalence and number of Shiga toxin-producing *Escherichia* (*E.*) *coli* (STEC) in animal feed was carried out over a period of nine years in the Republic of Croatia. A total of 1688 feed samples were collected from feed factories and poultry farms. Analysis included two standard procedures: sample enrichment and (a) immunomagnetic separation and plating on two selective media; or (b) plating on two selective media. Confirmation of STEC included morphological examination, biochemical tests, serotyping, and polymerase chain reaction. Morphological and biochemical characterization revealed 629 *E. coli* strains. Further serological screening method revealed 78 STEC and EPEC serotypes, while only 27 strains were confirmed as STEC with PCR. All positive samples (1.6%) originated from poultry farms and contained combination of virulence genes: *eaeA*, *stx1*, and/or *stx2*. Since the presence of *stx* (especially *stx2*) and *eae* are identified as risk factors for development of severe diseases in humans, results of this survey indicate that avian sources of STEC infections might be one of those “undefined sources” of human illnesses. Further research is necessary for evaluation of risks posed by contaminated feed, poultry, and environment.

## 1. Introduction

*Escherichia* (*E.*) *coli* is a heterogeneous group, comprising both pathogenic and non-pathogenic strains. Harmless commensals only occasionally (after they acquire additional virulence factors) cause diseases in vulnerable individuals (“YOPI”—young, old, pregnant and immunocompromised individuals). Certain strains are (highly) pathogenic and cause severe diseases in humans and animals [1,2,3,4,5,6,7]. Initially, differentiation of *E. coli* strains was made with serotyping, biochemical tests, and fermentative patterns. The first antigenic scheme for classification of *E. coli*, developed by Kauffmann, included 20 somatic (O) antigen groups. Currently, 173 O antigens, 80 K antigens, and 56 H antigens (with further diverging into partial antigens) are described, which gives approximately 50,000–100,000 different *E. coli* serotypes [8,9,10,11,12]. Although certain serotypes have been implicated in diseases in humans and animals, molecular studies did not confirm clear correlation between genetic relatedness and serotype of *E. coli* strain [7,13,14]. In addition, occurrence of non-typeable strains indicates that further research should apply several methodologies, especially when strains come from novel environments [15]. Genomes of pathogenic *E. coli* contain around 500–1000 more genes than the genome of non-pathogenic *E. coli* strain K12 MG1655 (4566 genes were found in this first complete *E. coli* genome sequence in 1997). Roughly, 2000 genes are considered common to all *E. coli* strains. It is considered that pathogenicity relies mainly on the presence of combination of virulence genes that are mainly clustered on pathogenicity islands, within bacteriophages and on plasmids [16,17,18,19,20,21]. Pathogenic *E. coli* strains are classified into pathotypes of zoonotic intestinal pathogenic *E. coli* (IPEC) and extraintestinal pathogenic *E. coli* (ExPEC). The later are associated with extra-intestinal infections (i.e., infections of urinary tract, septicemia, meningitis) in human host, and different diseases in animals (i.e., collibacilosis in poultry). Various serotypes share similar virulence genes (i.e., genes for antimicrobial resistance) between animal and human ExPEC and they can be classified as avian pathogenic *E. coli* (APEC), neonatal meningitis *E. coli* (NMEC), and uropathogenic *E. coli* (UPEC). The IPEC group (or diarrheagenic *E. coli* (DEC)) strains cause intestinal infections in human host that are characterized by mild to-severe (bloody) diarrhea, hemorrhagic colitis, hemolytic uremic syndrome, and sometimes even death. Six (sub) pathotypes are recognized: diffusely adherent *E. coli* (DAEC), enteroaggregative *E. coli* (EAggEC), enteroinvasive *E. coli* (EIEC), enteropathogenic *E. coli* (EPEC), enterotoxigenic *E. coli* (ETEC), and Shiga-toxin producing (Vero cytotoxin-producing) *E. coli* (STEC/VTEC), with a subgroup enterohemorrhagic *E. coli* (EHEC) [17,22,23,24,25,26,27]. The frequent gains and losses of genes due to different factors result in diversification of new strains, and sometimes “hybrid” pathotypes are described. Thus, characterization of strains with specific and/or non-specific combinations of virulence traits enable defining their pathogenic potential [28,29,30]. 

Shiga-toxin producing *E. coli* (or verotoxin-producing *E. coli*) produces one or more toxins (similar to cytotoxins of *Shigella dysenteriae*) that are known as Shiga toxins (also called Shiga-like toxins or verotoxins). Predominantly, toxins are produced by EHEC (STEC) group. There are two types of Shiga toxin in *E. coli*: Shiga toxin type 1 (Stx1) (equal to Stx of *S. dysneteriae*) and Shiga toxin type 2 (Stx2). They both have a number of subtypes (Stx1a, Stx1c, Stc1d, and Stx2a to Stx2g. Stx2h, Stx2i, Stx2k), and (nucleotide) variants (subtype, that differ by one or more amino acids from the prototype). Genes on prophages that are integrated into the chromosome [7,31,32,33] encode toxins. A significantly low dose of infection of STEC (fewer than 100 bacteria, and only ten cells for EHEC) with a short incubation period (2–5 days for *E. coli* O157:H7) emphasize the pathogenic power of this pathogen. In comparison, EPEC and ETEC infectious dose is ≥10^8^ CFU [34,35]. Mechanism that these pathogens use to invade the host and create an environmental niche for their propagation has been studied extensively. However, certain gaps in understanding still exist. For example, in the initial stage of the infection when the pathogen colonizes the gut, only intimin (94–97 kDA bacterial outer-membrane protein) has been described as the necessary bacterial adhesin for intimate attachment of pathogen to the host epithelial cells and causing “attaching and effacing (A/E)” lesions [36]. There are at least 17 antigenically distinct intimin subtypes [37] encoded by the *eae* gene and their importance has been documented in a number of infections. Since *eae* gene has not been detected in all STEC isolates, further research is necessary to elucidate this step of host infection [38,39,40,41]. Besides aforementioned production of Shiga toxins and intimin, there are a great number of additional virulence factors whose role has been studied in STEC infections, including autoagglutinating adhesin (Saa), catalase-peroxidase (katP), long polar fimbrial genes (lpf), plasmid-encoded enterohemolysin (ehxA), subtilase cytotoxin (subAB), and others.

In the latest report on the Zoonoses incidence in 2020 in the European Union, made by the European Food Safety Authority (EFSA) and European Centre for Disease Prevention and Control (ECDC), STEC infections were the fourth most reported zoonoses in humans, after campylobacteriosis, salmonellosis, and yersiniosis. A decrease in reported cases in 2020 was probably the result of COVID-19 pandemic, since the overall trend for STEC infections has been stable since 2016. The most common sources of human infections were food (meat of different types from different animal species, milk and dairy products and sometimes even fruits, vegetables, and other (not defined) sources). According to the available literature data on potential sources of human STEC infections, ruminants (particularly cattle) are considered as the main reservoir for STEC, while limited evidence exist about (wild) birds and other animals. Among reported serotypes, “top-five” serogroups were the most common (O26, O103, O111, O145, and O157). High rates of prevalence of STEC and diseases in humans have also been reported worldwide [42,43,44,45,46,47].

Since STEC is an important zoonotic pathogen, defining of the source of infection is crucial during outbreak investigations. Furthermore, presence of *stx* (especially *stx2*) and *eae* genes in bacterial strains are considered as risk factors for development of severe diseases in humans and animals. The aim of this research was to evaluate the role of animal feed as a potential source of this pathogen by determination of prevalence of STEC and determination of the presence of *stx* (and subtypes) and *eae* genes in isolates.

## 2. Materials and Methods

### 2.1. Samples Collection and Isolation of E. coli

In a nine year period (December 2012–November 2021), a total of 1688 feed samples were collected from feed factories and poultry farms situated in eight counties of the Republic of Croatia (Bjelovar-Bilogora County, Koprivnica-Križevci County, Krapina-Zagorje County, Požega-Slavonia, Sisak-Moslavina County, Zagreb County, Varaždin County and The City of Zagreb). All these areas are continental and cover around 30% of the country with around 45% of total population. Majority of feed factories and farms are located in this part of the country. Samples were either tested before use (directly from feed factory), or the samples have already been used for feeding of animals (sampled on poultry farms). Sampling has been done during each month of each year, which enabled determining the potential seasonality of pathogen isolation. Sampling of feed (dry or pelleted feed) to sterile container was made under aseptic conditions. Representative samples (25 g) were prepared according to guidelines for the preparation of test samples from laboratory samples of animal feeding stuffs (EN ISO 6498) and used for analysis. For detection of STEC, we have used two methods. Method (ISO 16654:2001) was employed for detection of *E. coli* O157:H7 and ISO/TS 13136:2012 method was used for detection of the other non-O157 STEC (Supplementary Materials, Figure S1).

The standard procedure for detection of *E. coli* O157 (ISO 16654:2001) enables detection of microorganisms that form typical colonies on cefixime tellurite sorbitol MacConkey agar (CT-SMAC) after incubation at 37 °C for 18–24 h. The procedure includes enrichment phase in modified tryptic soy broth (mTSB-N) with novobiocin (20 mg/L) and immunomagnetic separation for capturing *E. coli* O157 onto immunomagnetic particles (Dynabeads^®^ anti-*E. coli* O157, Applied Biosystems™, Oslo, Norway), followed by resuspending in 0.1 mL sterile buffer and plating of aliquot (50 µL) of particles/bacteria complex onto Sorbitol McConkey agar supplemented with cefixime tellurite in duplicate (CT-SMAC). Particles were also plated on the second (less) selective media, tryptone bile X-glucuronide agar (TBX). Five suspect sorbitol negative colonies (from CT-SMAC) were tested with confirmatory tests. Likewise, presumptive colonies (up to 50) were selected from TBX agar, pooled and tested with confirmatory tests. 

Adapted ISO/TS 13136:2012 was used for detection of other non-O157 STEC. Namely, the standard has been followed without screening part with real-time PCR and all samples were analyzed with culture methods for the presence of non-O157 STEC.

### 2.2. Characterisation of Isolated E. coli Strains

Confirmatory tests included latex agglutination test (*E. coli* O157 Latex, Oxoid, France) (detects O157 and H7 antigens), indole test, and API^®^ ID32E (bioMerieux, Marcy-l’Étoile, France). All isolates that were confirmed as *E. coli* with aforementioned confirmatory tests were plated on non-selective media and serotyped using polyvalent sera (SSI Diagnostica, Denmark, OK O pool 1—STEC/EPEC (O26, O103, O111, O145, and O157), 2—EPEC (O55, O119, O125ac, O127, and O128ab), 3—EPEC (O86, O114, O121, O126, and O142) antisera). The strains that showed agglutination with pool 1 antisera were further tested with monovalent antisera and analyzed by PCR. 

### 2.3. Identification and Characterization of Shiga Toxin-Producing E. coli Genes

Identification and characterization of STEC genes has been done by multiplex PCR method for detection of the main virulence genes: *eaeA*, *stx1*, *stx2*, *stx2f* (Table 1), according to the method developed by EU Reference Laboratory for *E. coli* (EURL-VTEC_Method_01_Rev 1) [48]. 

All strains that contained any of the aforementioned genes were also analyzed with the method that identifies three *stx1* subtypes (*stx1a*, *stx1c*, and *stx1d*) and seven *stx2* subtypes (*stx2a*, *stx2b*, *stx2c*, *stx2d*, *stx2e*, *stx2f*, and *stx2g*) by conventional PCR amplification). Shiga toxin producing *E. coli* (STEC) target genes, their primer sequences, and amplification fragment sizes for *stx* genes subtyping are presented in Table 2 (EURL-VTEC_Method_006_Rev 2) [48].

For the first method [48], a single bacterial colony was selected from the overnight culture of suspect bacteria on tryptone soy agar (TSA, Oxoid) and suspended in sterilized MilliQ water, boiled for ten minutes. A 50 µL reaction mixture (reaction buffer 1X, MgCl2 1.2 mM, dNTPs 0.2 mM each, 50 pmoles of each primer, 2 Uts of Taq polymerase (Promega) and 10 µL of DNA) was prepared for all samples, as well as for positive and negative control. For testing, we used the Thermal Cycler Biometra T3000 (Analytik Jena, Jena, Germany). Thermal profile included two minutes of initial denaturation and 35 PCR cycles: two minutes of denaturation at 95 °C; two minutes of annealing at 65 °C, decreasing to 60 °C by cycle 15; 1.5 min of elongation at 72 °C, increasing to 2.5 min from cycles 25 to 35, and final extension of three minutes [49]. Afterwards, each reaction mixture (18 µL), molecular weight marker (BenchTop 100 bp DNA ladder Promega ADG8291, Promega Cooperation, Madison, WI, USA), and loading die were loaded in a 2.0% (*w*/*v*) agarose gel in TE-buffer (10 mM Tris-HCL, 1 mM EDTA, pH 8) and run at constant voltage (100 V). The gel was stained with ethidium bromide and visualized under UV light. Samples that contained amplification fragments of the specific size (Table 1) were considered as positive for the corresponding target genes. 

The second step included subtyping method on all STEC isolates for which the presence of specific genes has already been assessed [48]. We have tested all the strains that show presence of *stx1* and/or *stx2* group genes. In short, a single bacterial colony was taken from overnight culture on TSA and inoculated in TSB. After overnight incubation, aliquot of 25 µL was mixed with 975 µL MilliQ water in an Eppendorf tube and boiled for 15 min. Afterwards, the samples were centrifuged (22.000 g/5 min) and transferred to a new clean tube. The supernatant was stored at −18 °C for further analyses. For PCR assay, we have prepared reaction mixture of 20 µL and additional 25 µL for triplex PCR. Reaction mixture included five µM of primers and five µL of supernatant of boiled lysate and filled up with MilliQ water to total volume of 20 and 25 µL. We have used HotStart Taq polymerase from Qiagen. Three different amplification conditions were used, each starting with 98 °C for 15 min (HotStart Taq activation) and ending with 72 °C for three minutes. First reaction condition was *stx1* subtyping (triplex PCR: 35 cycles at 94 °C for 50 s, 64 °C for 40 s and 72 °C for 60 s), second for *stx2a*, *b*, *c* subtyping (35 cycles at 94 °C for 50 s, 64 °C for 40 s and 72 °C for 60 s), and third for *stx2d*, *e*, *f*, and *g* subtyping (35 cycles at 94 °C for 50 s, 64 °C for 40 s, and 72 °C for 60 s). For each reaction, we used appropriate positive and negative control. The samples were loaded in 2.0% (*w*/*v*) agarose gel in TE-buffer (10 mM Tris-HCL, 1 mM EDTA, pH 8) at quantity of 18 µL of reaction along with loading dye (1X final concentration). The gel was run at a constant voltage (100 V). For evaluation of produced amplicons, we have used molecular weight marker. Visualization of results was made after staining with ethidium bromide under UV light. All procedures have been done using adequate protective equipment. Evaluation of tested isolates included analysis of prevalence of serotypes, seropathotypes, and defining of the discriminatory power of subtyping of STEC with respect to O antigen and corresponding seropathotypes by calculation of Simpson’s index of diversity [50,51].

## 3. Results

### 3.1. Isolation and Characterisation of Isolated E. coli Strains

**Figure 1:**
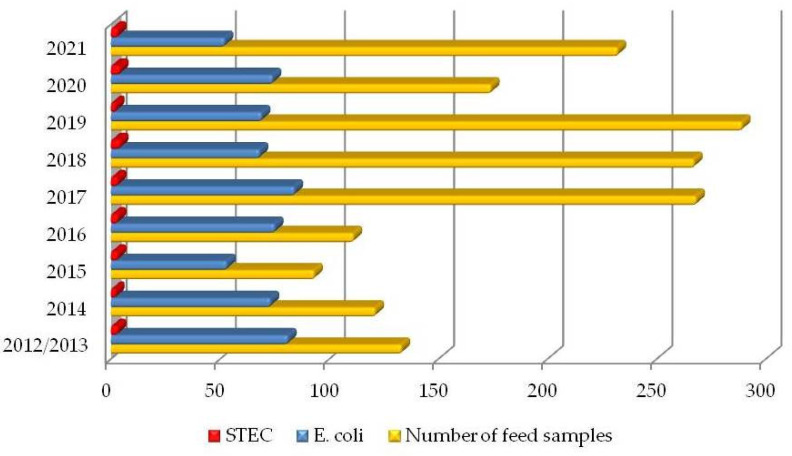
Incidence of *E. coli* and STEC (confirmed at least one strain by detection of *stx* genes) in analyzed feed samples in a nine-year period (December 2012–November 2021).

Morphological and biochemical characterization showed the presence of *E. coli* in 629 (37.3%) feed samples originating from all tested areas in Croatia with a slight seasonal effect (highest in July–August). Further analysis with serological screening method revealed 78 STEC and EPEC serotypes (12.4%) (Figure 1). Despite characteristic growth on selective media and biochemical tests of 551 isolates, they were not confirmed as STEC by using specific antisera and were not further tested with PCR. 

### 3.2. Serotypes and Seropathotypes of Isolated STEC Strains

**Figure 2:**
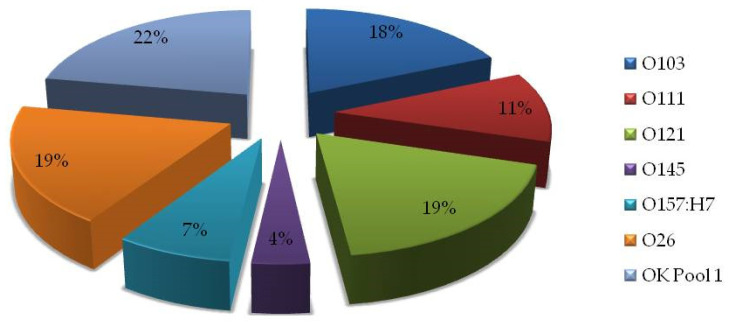
Frequency of serotypes of isolated STEC from feed in Croatia during the nine-year period (*n* = 27 isolates). OK Pool 1: not fully typed, isolates gave positive agglutination with OK Pool 1, but not with individual antisera (*n* = 7).

Among 78 isolated STEC and EPEC serotypes, only 27 samples (4.3% of analyzed strains and 1.60% of all samples tested) were serotyped as positive with OK Pool 1 antisera. All positive samples (*n* = 27) originated from poultry farms. Remaining 51 isolates gave positive agglutination results with OK Pool 2 (*n* = 23) and OK Pool 3 (*n*= 28) antisera. Among 27 STEC-positive isolates, we have detected following serotypes: O26, O103, O111, O121, O145, and O157:H7. Seven isolates did not show agglutination with any of the monovalent sera, although OK Pool 1 reaction was positive (Figure 2). 

**Figure 3:**
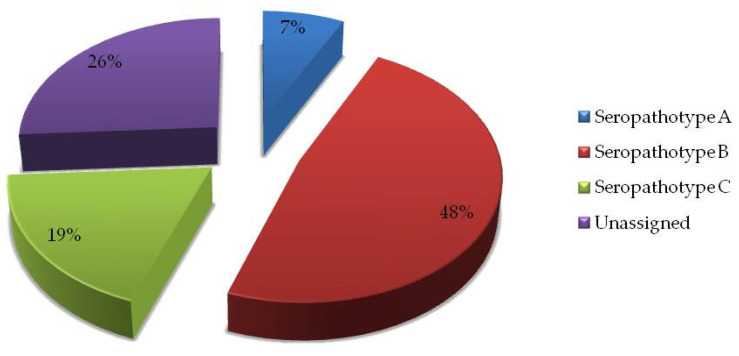
Distribution of seropathotypes (SPT) of isolated STEC from feed during the nine-year period (*n* = 27 strains): SPT A: *n* = 2; SPT B: *n* = 13; SPT C: *n* = 5; Unassigned: not fully typed (*n* = 7), seropathotypes are defined by Karmali et al. [27].

Seropathotypes (SPT) are defined by Karmali et al. [27] as an approach to classify pathogenic *E. coli* which have been associated with the onset of human disease. Namely, SPT A has high incidence, it is common in outbreaks, and is associated with HUS or hemorrhagic colitis in humans; SPT B corresponds to moderate incidence, it is uncommon in outbreaks, but is also associated with HUS or hemorrhagic colitis cases in humans; SPT C characterizes low incidence, it is rare in outbreaks, but is also associated with HUS or hemorrhagic colitis in humans. Evaluation of seropathotypes (SPT) of isolated STEC showed that the majority (48%) of isolates belong to SPT B, characterized by moderate incidence, uncommon in outbreaks, but associated with HUS or HC in human hosts. Only 7% belong to SPT A that is characterized with high incidence, common in outbreaks, and associated with HUS or HC in humans (Figure 3). 

Evaluation of discriminatory power of subtyping of STEC with respect to O antigen and corresponding seropathotypes isolated in our study was made by calculation of Simpson’s index of diversity. The index, originally developed for the ecological studies of intraspecies populations, is used for determination of the probability that two unrelated strains, sampled from the test population, are placed in different typing groups. The overall serotype diversity in our study was 0.86 for antigen typing method and 0.69 for classification of seropathotypes.

### 3.3. Virulence Profile of Positive STEC Strains (No = 27)

All isolates that were serotyped positive with OK Pool1, OK Pool2, and OK Pool3 were analyzed by PCR (Table 3). Among 78 isolated strains, only 27 samples (1.60%) harbored at least one of the tested genes (*eaeA*, *stx1*, and/or *stx2*). All positive samples (*n* = 27) originated from poultry farms. Remaining 51 isolates did not harbor tested genes.

## 4. Discussion

The importance of animals as a potential source of human infection is evident in the fact that STEC strains (with similar virulence characteristics) have been isolated from human patients, apparently healthy animals, meat, and products thereof. Majority of research on prevalence of STEC in animals and food is focused on ruminants since they are considered as their natural reservoir, although there are several reports on prevalence in other animals and their products [52,53,54]. For example, Bai et al. [55] reported finding of STEC in retail meat samples of pork (4.4%), beef (11.0%), mutton (20.6%), chicken (0.5%), and duck (7.7%), as well as in healthy pigs and yaks. In the latest European Union Zoonoses Report, during 2010–2019, strong-evidence STEC outbreaks were traced to bovine meat and products thereof, water, vegetables and juices, and other products thereof, milk and cheese [56]. In a case of sporadic cases, “strong evidence” was rarely possible to define. Generally, it is observed that STEC contamination of food was 2.5%. When testing fresh meat, the same report showed incidence of STEC in 1.6% of bovine meat, 11.4% of ovine meat, 7.7% of pig meat, and 4.2% in meat from other animal species that included broilers, ducks, wild and farmed game, geese, horses, poultry, rabbits, turkeys, wild boars, and unspecified meat. In animals, observed frequency of STEC was documented for cattle (5.2%), sheep and goats (5.5%), pigs (48%) and other animals that included Cantabrian chamois, deer, wild boar, water buffalo, birds, and foxes (3.2%) with overall incidence of 6.3%. However, all these data should be evaluated as estimates since the number of samples is rather low and applied methodologies are not always comparable. In addition, a number of epidemiological research did not result in defining the source, indicating the need for research on other potential sources of human infections that should also include various animal species including poultry.

Recent finding of pet food positive chicken meat highlights the importance of (re)evaluation of poultry as well as other sources of STEC infections in animals and humans [52]. Furthermore, there is limited information on the detection of STEC and their serogroups in animal feed and especially in feed intended for poultry. Although only a few studies have addressed this issue, number of tested samples and non-comparable methodologies cannot rule out the possibility of feed and avian sources of human infections, especially when poultry meat is still the staple food of many healthy diets. Our results showed low incidence of STEC strains in feed samples derived from poultry farms. Since the sampling strategy was random and did not include additional samples (environment, poultry, personnel) it is difficult to trace the origin of these isolates. However, finding of potentially harmful strains highlights the importance of study at the level of poultry farms. Seasonality of *E. coli* isolates has already been well documented and it has been described for human STEC illness outbreaks. Although a number of analyzed samples were not equally distributed during the tested time period, we have noticed similar seasonal trend (higher incidence in summer months in our study) as the one that was reported for human illness cases in the EU countries in the period between 2010 and 2020 [43,53,54]. With respect to annual variation, our study shows higher prevalence in the period from 2012 and 2016 year. One potential explanation could be the number of analyzed samples, showing that higher number of analyzed samples yields results that are more precise. On the other hand, it is well-known that *E. coli* and pathogenic *E. coli* are widespread in different environments and that they have the ability to persist diverse (un)favorable conditions. A number of different factors including environment, other hosts, and procedures in the feed/food-production-chains might influence the final rates of STEC that we have noticed in our study. Therefore, these results indicate that future research is needed to generate comparable data on (seasonal and annual) prevalence and trends of STEC in feed, animals, and environment.

Incidence of STEC-positive isolates in our study (1.60%, 27 positive of 1688 samples tested) is lower than the reported prevalence of STEC in 2020 in the European Union in fresh meat samples taken from broilers, ducks, wild and farmed game, geese, horses, poultry, rabbits, turkeys, wild boards, and unspecified meat (4.2%, seven positive of 166 samples tested). Since the sample type is not the same, this might explain the noted difference. Previous reports on incidence of STEC in feed and avian sources by other authors show no or rather low incidence (0.5 to 15.56%), although it is not uncommon that avian *E. coli* harbors *stx* genes [6,54,55,56,57,58,59,60,61,62,63,64]. Although the STEC prevalence levels in our study and other reports are low, and the fact that only samples from the farm were positive, the transmission of STEC to other birds and environment present a potential risk. Animals are usually asymptomatic carriers making the route of dissemination of pathogens hard to trace. Namely, for some wild birds it has been reported that STEC O157 can be present in their feces for up to 13 days post colonization at the levels above 100 CFU/g and thus transmit infection to other animals and humans. For chickens, up to 11 months of shedding of STEC O157 has been reported [65,66,67]. 

Growth and survival of pathogenic *E. coli* in host and environment is influenced by different factors including their abilities to acquire nutrients and energy sources as well as mechanisms that bacteria use to overcome various stressful conditions. Whether environmental fitness of STEC is directly correlated with their pathogenicity can be explained if adaptation mechanisms are investigated along with its gene expression patterns. In addition, persistence in the environment in a form of “dormant” cells maybe does not mean a real threat. However, eliminating the potential sources (animals, feed, food, environment, etc.,) by application of appropriate preventive measures will, at the end, result in a lower risk of infection. Furthermore, it is important to define to what extent can STEC present a threat for infections in human, when poultry industry is in question. Previous research has shown that ruminants are reservoirs while other animals are actually “amplifying hosts”. Presence of STEC in poultry meat points out on poultry as the source. However, it is still not clear what is the relevant contribution and significance of poultry for STEC infection in humans. According to the research of Munghini-Gras et al. [68], estimated contribution of poultry is only 3% but it is not clear whether poultry is the actual host or is it the result of the exposure to contaminated environment. Careful examination of all potential routes of infection (including water, feed, environment and humans) and application of case-control and source attribution analyses would enable proper risk assessment in this area of research.

With respect to serogroups, data are also not completely comparable, since the data on serogroup were not reported for all tested samples (no = 1981) in EU report. Namely, of 54 positive samples, only 11 samples included data on serogroup and those included O6, O8, O104, O11, O145, O146, and O157. Two detected serogroups (O111 and O145) belong to the “top-five” serogroups that are considered responsible for 70–80% of non-O157 STEC produced illnesses. Five serogroups (O8, O111, O145, O146, and O157) detected in this study belong to “top-20” of the most frequent serogroups reported in confirmed cases of human STEC infections and of STEC in food and in animals in the EU in 2020. In our research, four serogroups (O26, O103, O111, and O145) belong to the most common non-O157 serogroups associated with diseases in human in Europe, while O121 and O157 belong to “top-20”. It is interesting to note that the most common non-O157 serogroups in the USA are O26, O45, O103, O111, O121, and O145. Unfortunately, we cannot trace the source of STEC in our samples, nor define impact of feed ingredients and their origin, especially because all the samples originated from Feed Factories were negative. We can only speculate on potential environmental sources or even contact with other animals and/or shedding by asymptomatic workers [47]. In addition, further characterization of the strains with the same serotype is necessary. Therefore, future research will include evaluation of serotypes, virulence factors and defining whether the isolated strains are clonally related. 

Similar comparison problem with the data from EFSA’s report is also with virulence profile. Namely, information on virulence profile of STEC isolates was reported only for 13 STEC isolates. Among them, nine were *stx2*+ and four were *stx1*+, while *eae* gene was either negative or this information was also not provided [56]. In our results, all positive STEC isolates harbored at least one tested gene (*eaeA*, *stx1*, and/or *stx2*). However, we have also detected higher incidence of *stx2*+ genes than *stx1*+, and presence of *eae* gene in all STEC-positive isolates. Absence of *eae* gene in STEC positive strains has been described by other authors, as well as potential loss of specific genes after infection and/or subcultivations [69,70]. Furthermore, presence of *eae* gene in our strains implicate that they might be more prone to intimate attachment to epithelial host cells and causing “attaching and effacing (A/E)” lesions [36]. According to several authors, evaluation of avian sources of pathogenic *E. coli* is by defining of EPEC groups. Thus, EPEC was divided into two groups, typical (tEPEC) and atypical (aEPEC) with respect to presence or absence of EPEC adherence factor plasmid (pEAF), respectively. Such strains have also been isolated from avian sources. A number of other features that differentiate those groups have also been described [71,72,73,74]. Following this classification, further research on the presence of EPEC Adherence Factor plasmid and other virulent traits are necessary to evaluate whether there are any relatedness of our strains with the EPEC group. With respect to diversity of isolated STEC in our study, high SDI index for serotyping (0.86) showed high serotype diversity. Taking into account the limits for determination of diversity based on serotyping methods, determination of true diversity can be done using sample-serotype-virulotype profiles. This has also confirmed the importance of simultaneous application of several methodologies, especially in epidemiological studies. Finally, evaluation of poultry as the potential source of STEC strains will be possible only by applying a combination of comparable detection methods and defining the responsible vehicles of infection (with detailed information on contributory factors and source). Evaluation of seropathotypes (SPT), as described previously [27], of isolated STEC showed that 81% (SPTA + SPTB + SPTC) could be associated with human infections such as HUS or HC. Although only 7% belong to SPTA (high incidence and common in outbreaks), feed and/or poultry might be either source or vehicle of potential human infections. 

## 5. Conclusions

Certain serotypes have been implicated in diseases in humans and animals, although molecular studies did not confirm direct correlation between genetic relatedness and serotype of *E. coli*. Besides defined serotypes and seropathotypes, we have also detected several non-typeable strains for which specific STEC genes were confirmed. Considering the fact that there is a number of other potential virulence traits, as well as the constant emergence of new pathotypes due to the frequent gains and losses of genes, future research needs to include several methodologies, especially when strains come from novel environments. Characterization of isolated STEC strains with specific and/or non-specific combinations of virulence traits, as well as evaluation of other virulence factors will enable defining of their pathogenic potential. Results of this study indicate that avian sources of STEC might be one of those “undefined sources” of human illnesses. Further research will provide necessary data on the role of feed, environment, poultry, and other avian sources in epidemiological studies and aid in evaluation of pathogenic potential of STEC infections in humans and animals.

## Figures and Tables

**Table 1:** Shiga toxin producing *E. coli* (STEC) target genes, their primer sequences and amplification fragment sizes for multiplex PCR reaction method developed by EU Reference Laboratory for *E. coli* (EURL-VTEC_Method_01_Rev 1) [48].

Target Gene	Primer Sequence	Amplicon Size (bp)
*eaeA*	*eaeAF* (GACCCGGCACAAGCATAAGC) *eaeAR* (CCACCTGCAGCAACAAGAGG)	384
*stx1*	*stx1F* (ATAAATCGCCATTCGTTGACTAC) *stx1R* (AGAACGCCCACTGAGATCATC)	180
*stx2*	*stx2F* (GGCACTGTCTGAAACTGCTCC) *stx2R* (TCGCCAGTTATCTGACATTCTG)	255
*stx2f*	*stx2fF* (AGATTGGGCGTCATTCACTGGTTG) *stx2fR* (TACTTTAATGGCCGCCCTGTCTCC)	428

**Table 2:** Shiga toxin producing *E. coli* (STEC) target genes, their primer sequences and amplification fragment sizes for gene subtyping method developed by EU Reference Laboratory for *E. coli* (EURL-VTEC_Method_006_Rev 2) [48].

Target Gene	Primer Sequence (5′–3′)	Amplicon Size (bp)
***stx1***		
***stx***1a-F1 ***stx***1a-R2	CCTTTCCAGGTACAACAGCGGTT GGAAACTCATCAGATGCCATTCTGG	478
***stx***1c-F1 ***stx***1c-R1	CCTTTCCTGGTACAACTGCGGTT CAAGTGTTGTACGAAATCCCCTCTGA	252
***stx***1d-F1 ***stx***1d-R2	CAGTTAATGCGATTGCTAAGGAGTTTACC CTCTTCCTCTGGTTCTAACCCCATGATA	203
***stx2***		
***stx***2a-F2 ***stx***2a-R3 ***stx***2a-R2	GCGATACTGRGBACTGTGGCC CCGKCAACCTTCACTGTAAATGTG GGCCACCTTCACTGTGAATGTG	349 347
***stx***2b-F1 ***stx***2b-R1	AAATATGAAGAAGATATTTGTAGCGGC CAGCAAATCCTGAACCTGACG	251
***stx***2c-F1 ***stx***2c-R2	GAAAGTCACAGTTTTTATATACAACGGGTA CCGGCCACYTTTACTGTGAATGTA	177
***stx***2d-F1 ***stx***2d-R1 ***stx***2d-R2	AAARTCACAGTCTTTATATACAACGGGTG TTYCCGGCCACTTTTACTGTG GCCTGATGCACAGGTACTGGAC	179 280
***stx***2e-F1 ***stx***2e-R2	CGGAGTATCGGGGAGAGGC CTTCCTGACACCTTCACAGTAAAGGT	411
***stx***2f-F1 ***stx***2f-R1	TGGGCGTCATTCACTGGTTG TAATGGCCGCCCTGTCTCC	424
***stx***2g-F1 ***stx***2g-R1	CACCGGGTAGTTATATTTCTGTGGATATC GATGGCAATTCAGAATAACCGCT	573

**Table 3:** Serotypes and virulence profile of isolated STEC in analyzed feed samples in a nine-year period (December 2012–November 2021).

Isolate	Serotype	STEC Target Genes		
		*eaeA*	*stx*1	*stx*2
1	O26	+	-	+(*stx*2a)
2	O145	+	+(*stx*1c)	+(*stx*2a)
3	O121	+	-	+(*stx*2a)
4	O111	+	+(*stx*1a)	-
5	O103	+	+(*stx*1a)	-
6	O111	+	+(*stx*1a)	-
7	O157:H7	+	-	+(*stx*2a, *stx*2c)
8	O26	+	-	+(*stx*2a)
9	OK Pool 1	+	-	+(*stx*2a)
10	O121	+	-	+(*stx*2a)
11	O26	+	-	+(*stx*2a)
12	O103	+	+(*stx*1a)	-
13	OK Pool 1	+	-	+(*stx*2a)
14	O103	+	+(*stx*1a)	-
15	O157:H7	+	-	+(*stx*2a, *stx*2c)
16	OK Pool 1	+	-	+(*stx*2a)
17	O121	+	-	+(*stx*2a)
18	O103	+	+(*stx*1a)	-
19	OK Pool 1	+	-	+(*stx*2a, *stx*2c)
20	O121	+	-	+(*stx*2a)
21	O26	+	+(*stx*1a)	+(*stx*2a)
22	O121	+	-	+(*stx*2a)
23	OK Pool 1	+	-	+(*stx*2c)
24	O103	+	+(*stx*1a)	-
25	OK Pool 1	+	-	+(*stx*2a, *stx*2c)
26	O111	+	+(*stx*1a)	-
27	OK Pool 1	+	+(*stx*1a)	+(*stx*2a)

Note: OK Pool 1—if no agglutination was noticed with individual sera.

## Data Availability

Not applicable.

## Supplementary Materials

The following supporting information can be downloaded at: www.mdpi.com/article/10.3390/microorganisms10091839/s1.
