# Supplementary material for: Prevalence and Characterization of Shiga Toxin Producing Escherichia coli Isolated from Animal Feed in Croatia
Source: Microorganisms. 2022 Sep 14;10(9):1839. doi: 10.3390/microorganisms10091839 (PMC9505133; doi:10.3390/microorganisms10091839)
Supplement: Supplementary file 1 [file microorganisms-10-01839-s001.zip › microorganisms-1865265-supplementary.pdf]

Supplementary Materials. Figure S1. Flow diagram of the procedures for isolation and characterization of STEC from feed samples 37 °C ± 1 °C

25 g of Feed Sample

↓

Isolation of bacteria

ISO 16654<br>*For detection of E. coli O157*	ISO/TS 13136<br>*For detection of non-O157 STEC*
Enrichment in mTSB-N pre-warmed to 41.5 °C	Enrichment in mTSB-N pre-warmed to 41.5 °C (or in BPW pre-warmed to 37 °C)
Homogenization and incubation for 6 h then a further 12 h to 18 h at 41.5 °C	Homogenization and incubation in mTSB-N for 18 h to 24 h at 41.5 °C (or BPW for 18 to 24 h at 37 °C)

↓

Concentration of *E. coli* O157 by capture onto immunomagnetic particles and washing with sterile wash buffer	Inoculation of 50 µl onto selective medium
Re-suspension in 0,1 ml of sterile wash buffer	
Inoculation of 50 µl of the washed and re-suspended magnetic particles on selective medium to obtain isolated colonies	

↓

CT-SMAC<br>Incubation at 37 °C for 18 h to 24 h	TBX<br>Incubation at 37 °C for 18 h to 24 h
Selection of 5 typical colonies (sorbitol negative)	Selection of 5 presumptive colonies

Confirmatory tests

Confirmation of pure colonies by indole formation (or other biochemical identification kit)

Agglutination test for E. coli O157 Latex Test for detection of O157 and H7 antigens	Confirmation by serological identification with polyvalent antisera OK O pool 1, 2, 3 and monovalent OK O pool 1 antisera (O26, O103, O111, O145 and O157)
Confirmation by serological identification with antiserum to *E. coli* O157	

Detection of main virulence genes

DNA extraction of single/or pool colonies (selected from up to 50 confirmed colonies after enrichment broth and growth on suitable solid media)

↓

Multiplex PCR method for detection of main virulence genes: *eaeA, stx1, stx2, stx2f* (*EURL-VTEC_Method_01_Rev* 1), If positive for at least one virulence gene, isolates are subjected to second PCR method to define subtypes

↓

Multiplex PCR method that identifies three *stx1* subtypes (*stx1a, stx1c,* and *stx1d*) and seven *stx2* subtypes (*stx2a, stx2b, stx2c, stx2d, stx2e, stx2f* and *stx2g*) by conventional PCR amplification (*EURL-VTEC_Method_006_Rev* 2)

Results reporting

Notes: *CT-SMAC – Cefixime Tellurite Sorbitol MacConkey Agar; mTSB-N – modified Tryptic Soy Broth with 20 mg/L of Novobiocin; Immunomagnetic particles Dynabeads® anti-E. coli O157 (Oxoid or equivalent); TBX – Tryptone Bile X-glucuronide agar, used as second (less) selective media; OK O pool 1 - STEC/EPEC (O26, O103, O111, O145 and O157), 2 - EPEC (O55, O119, O125ac, O127 and O128ab), 3 - EPEC (O86, O114, O121, O126 and O142) antisera (SSI Diagnostica) or equivalent.*
